# How Effective Are Dietitians in Weight Management? A Systematic Review and Meta-Analysis of Randomized Controlled Trials

**DOI:** 10.3390/healthcare7010020

**Published:** 2019-02-01

**Authors:** Lauren T. Williams, Katelyn Barnes, Lauren Ball, Lynda J. Ross, Ishtar Sladdin, Lana J. Mitchell

**Affiliations:** Menzies Heath Institute Queensland, Gold Coast Campus, Griffith University, Parklands Dr, Southport, QLD 4222, Australia; k.barnes@griffith.edu.au (K.B.); l.ball@griffith.edu.au (L.B.); lynda.ross@griffith.edu.au (L.J.R.); i.sladdin@griffith.edu.au (I.S.); lana.mitchell@griffith.edu.au (L.J.M.)

**Keywords:** dietitian, workforce, dietetic consultation, nutrition care, nutritional management, primary health care

## Abstract

Effective, evidence-based strategies to prevent and treat obesity are urgently required. Dietitians have provided individualized weight management counselling for decades, yet evidence of the effectiveness of this intervention has never been synthesized. The aim of this study was to examine the effectiveness of individualized nutrition care for weight management provided by dietitians to adults in comparison to minimal or no intervention. Databases (Cochrane, CINAHL plus, MedLine ovid, ProQuest family health, PubMed, Scopus) were searched for terms analogous with patient, dietetics and consultation with no date restrictions. The search yielded 5796 unique articles, with 14 randomized controlled trials meeting inclusion criteria. The risk of bias for the included studies ranged from unclear to high. Six studies found a significant intervention effect for the dietitian consultation, and a further four found significant positive change for both the intervention and control groups. Data were synthesized through random effects meta-analysis from five studies (n = 1598) with weight loss as the outcome, and from four studies (n = 1224) with Body Mass Index (BMI) decrease as the outcome. Groups receiving the dietitian intervention lost an additional 1.03 kg (95% CI:−1.40; −0.66, *p* < 0.0001) of weight and 0.43 kg/m^2^ (95% CI:−0.59, −0.26; *p* < 0.0001) of BMI than those receiving usual care. Heterogeneity was low for both weight loss and BMI, with the pooled means varying from 1.26 to −0.93 kg and −0.4 kg/m^2^ for weight and BMI, respectively, with the removal of single studies. This study is the first to synthesize evidence on the effectiveness of individualized nutrition care delivered by a dietitian. Well-controlled studies that include cost-effectiveness measures are needed to strengthen the evidence base.

## 1. Introduction

Obesity is acknowledged as a problem of pandemic proportion. The public health efforts of the past three decades have not only failed to reverse population weight gain, but have not even managed to slow the rate of increase [1]. In most Western countries, more than half of the adult population has a Body Mass Index (BMI) above the healthy weight range, which places stress on health care systems due to the concomitant increases in incidence of obesity-related conditions such as Type 2 Diabetes Mellitus (T2DM) [2]. Given that the proportion of the adult population who could benefit from prevention is now the minority, intervention efforts need to be aimed at the majority, which, in western countries, is the treatment of those already overweight or obese.

Clinical counselling focused on dietary change is one of the key strategies identified for the treatment of adult obesity [3]. Dietitians are health professionals with specialized training in weight management, and are recognized as the key professional group to provide this treatment [4]. Individualized dietetic care for weight management is largely provided in the outpatient or primary health care setting since it typically occurs with the chronic diseases treated in this setting. Dietitians take a standardized approach to treating individualized clients through the dietetic consultation, which follows the structured Nutrition Care Process of nutrition assessment, nutrition diagnosis, nutrition intervention and nutrition monitoring and evaluation [5].

Professional associations of dietitians, such as The Academy of Nutrition and Dietetics in the USA, provide guidelines to members for the management of obesity in patients [6]. As the nutrition experts of the healthcare workforce, dietitians have a particular responsibility for implementing these guidelines. The position paper of the Academy on interventions for the treatment of overweight and obesity in adults, updated in 2016 to reflect new evidence, recommends that dietitians ‘provide expertise in the area of nutrition’ in the management of these patients [6] (p. 142). The British Dietetic Association (BDA) has recently released an updated report on the evidence and clinical application for dietetic obesity interventions in adults [7]. The BDA’s work acknowledges and is based on guidelines developed by other agencies [8,9] but is deliberately focused on the role of the dietitian in providing an individual dietetic consultation ‘given this is the most common form of dietetic contact’ [7] (p. 4). Similarly, in 2013, the Dietitians Association of Australia (DAA) published a set of best-practice guidelines for the treatment of overweight and obesity in adults [10]. These guidelines were constructed from evidence provided by a series of systematic reviews of weight control interventions and were designed to inform the practice of dietitians. 

These, and other national and international authorities such as the World Health Organization have reviewed the evidence on effective interventions for overweight and obesity for adults and set desired intervention goals. Given the relative abundance of systematic reviews and evidence-based guidelines on obesity management, including those published by dietetic associations, it is surprising and somewhat concerning that the particular contribution made by dietitians to the management of obesity remains unknown. While reviews have recently been published on dietitian effectiveness in T2DM treatment and prevention [11,12], data on dietitian effectiveness in obesity management have not previously been synthesized. Our recent systematic review collected and qualitatively synthesized all the papers published on dietetic consultations for a variety of patient outcomes including weight management [13]. The purpose of this current paper was to quantify the effectiveness of dietitians providing individualized nutrition care to adults for weight control through a meta-analysis of relevant studies. 

## 2. Materials and Methods

### 2.1. Systematic Review

Detailed methods of the systematic review conducted to address the question of effectiveness of the dietetic consultation for all types of patient outcomes in the primary health setting have been published elsewhere [13] with relevant details summarized here. The review focused on studies with a randomized controlled trial (RCT) design in order to synthesize the highest level of evidence. Of the studies that met the eligibility criteria, only those directed at interventions for weight control (weight loss or weight gain prevention) were included in the current review. The studies needed to have included weight control as a stated study aim and/or specified it as a primary outcome measure and/or specified weight loss or weight gain prevention information as part of the intervention advice. Studies that collected anthropometric data but did not include a focus on weight control in the nutrition care provided by dietitians were excluded. Meta-analysis was further restricted to those studies that reported data in a form compatible with meta-analysis. 

The inclusion criteria for the review are given in Table 1 [13]. Articles were excluded if they did not report on the outcomes of the interventions delivered by the dietitians exclusively (for example if delivered by a multi-disciplinary team), or if the interventions were conducted in a group format or in a hospital, rather than primary health care setting. If multiple active intervention arms were included, only the dietetic consultation arm data were extracted.

The literature search was conducted on journal articles published up to October 2016 in the ProQuest Family Health, Scopus, PubMed Central, MEDLINE^®^, CINAHL, and Cochrane databases. Keywords were combined using the Boolean operators ‘AND’ and ‘OR’ to create the following three search categories: ‘patient OR client OR client-centered OR participant OR adult’ AND ‘dietitian OR dietetic’ AND ‘consult* OR referral OR practice OR counselling OR interview OR advice OR outpatient OR clinic’. Limits included humans, adults and English language. There were no date restrictions to the search, given that the body of literature had not previously been synthesized. The original search was conducted in October 2016 and an updated search was conducted in October 2018. Each abstract was screened in duplicate. The reference lists of the systematic reviews that did not themselves meet the eligibility criteria were searched for eligible articles.

The full text of all the articles that met the inclusion criteria were retrieved and examined by two researchers. Two researchers independently assessed the quality of each of the included publications using the Cochrane Risk of Bias tool. Data from the included publications were extracted in duplicate. Publications reporting data on anthropometry outcomes were examined and the following data were extracted: the study identification (first author, year, country), primary study aim, recruitment criteria, participant characteristics at baseline (gender, age, adiposity), sample size and numbers completing (intervention and comparator), analysis technique (intention-to-treat (ITT) or per-protocol analysis), study end point, description of the dietitian intervention (type of dietary advice, frequency, duration), comparator (usual, minimal or no intervention), and risk of bias. For the outcomes, data on the measurement methods (self-report of measured) and the measures reported (weight, waist, percent body fat and other metabolic measures of glycemic control and blood pressure) at baseline and at the end of the intervention period were extracted, noting whether the outcomes were stated as primary or secondary. The mean values and variance measures (standard deviation (SD) and standard error of the mean (SEM) or 95% confidence intervals (CI)) were extracted. All variance measures were converted to standard deviations using the Cochrane Handbook method [14]. The weight data reported in pounds were converted to kilograms using a standardized conversion (divide pounds by 2.206). 

### 2.2. Meta-Analyses

Meta-analyses were conducted using Revman version 5.3 [15]. The studies included in the review were only eligible for meta-analysis if they stated weight or BMI as part of their study aim, and if they reported adequate data for the calculation of the absolute mean change in the anthropometric measure from baseline to the end of the intervention. When the mean change and variance was not reported, it was calculated based on the difference between means and the point SD was calculated from the 95% confidence interval (CI) [14]. The medians and interquartile range (IQR) data were not excluded. Rather, for studies that had greater than 100 participants, the range was estimated [16] and then used to estimate SD [17]. The meta-analyses of interventions aimed at preventing weight gain were conducted separately from the meta-analysis of interventions focused on weight loss. Pooled effect sizes were calculated as the weighted mean difference (WMD) and 95% CI for absolute change in measures. Heterogeneity was quantified using the I^2^ statistic values of approximately 25%, 50% and 75% considered to indicate low, moderate and high-level of heterogeneity, respectively. A random effects model was used due to the high heterogeneity of the dietetic interventions and the complexity of individual variability in response to lifestyle modification [18]. Sensitivity analyses were conducted using the “leave-one-out” method, to assess whether any single study elicited undue influence on the overall result. 

## 3. Results

### 3.1. Characteristics of Included Studies

**Figure 1:**
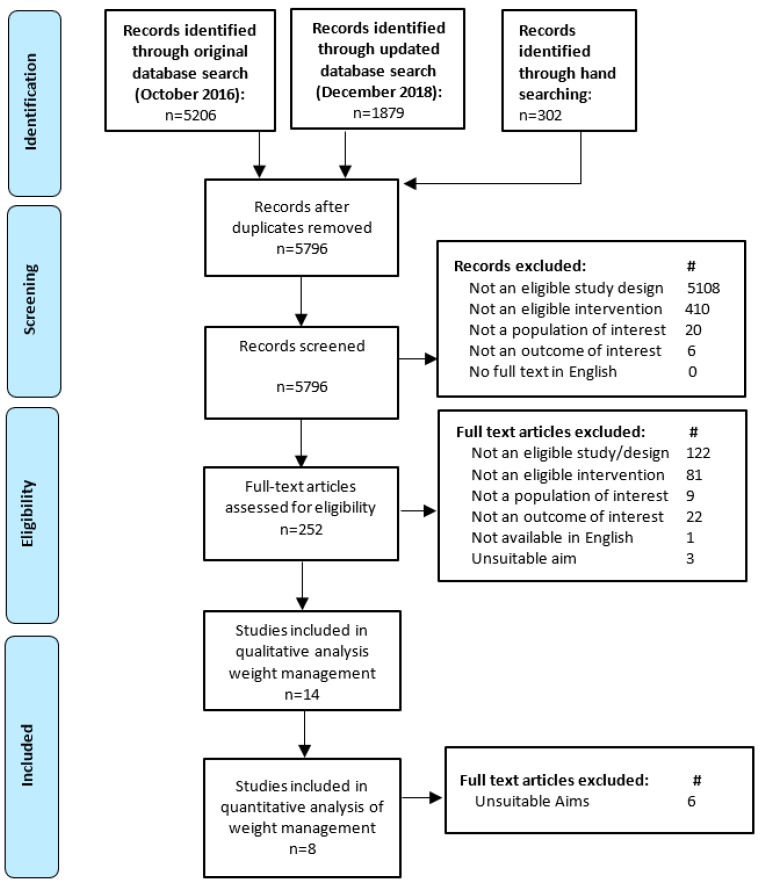
Flow diagram of the literature search and filtering results for a systematic review of the effectiveness of individual dietetic consultations for managing weight.

Figure 1 outlines the results of the literature search and screening processes. No systematic reviews were found that met the inclusion criteria. Fourteen RCT studies were eligible for inclusion in the review. The characteristics and outcomes of those studies are given in Table 2 according to the study aims. No studies were rated as having a low risk of bias. Seven had an unclear rating [19,20,21,22,23,24,25] and the remaining seven [26,27,28,29,30,31,32] had a high risk of bias. The main cause of the high risk of bias was the rate of participant dropout resulting in incomplete long-term data. Other common sources of bias included inadequate reporting for participant blinding, blinding of outcome assessment, allocation concealment, and sequence generation. Seven of the studies calculated the effect for their primary outcome variables using intention-to-treat (ITT) principles [19,20,21,22,24,25,28], three of which also reported per-protocol analyses [21,25,28]. For those studies reporting ITT results, last observation carried forward (LOCF) was the most common approach to handling missing data [21,24,28]. Six studies [23,27,29,30,31,32] relied only on per-protocol analysis to assess the intervention effects. The study by Imai and colleagues made no mention of whether ITT analysis was used. However, the tables appear to state that there were no dropouts during the 12 months of the study [26]. 

The oldest included study was published in 1978 [30] and the most recent was published in 2018 [25], with the majority of the studies published since the year 2000. Four of the studies were conducted in the USA [20,23,28,29], two in Australia [19,27], two in China [24,25], and the remainder in Denmark [31], Italy [21], Japan [26], Scotland [30], Taiwan [32] and one trial was multinational (nine countries) [22]. While the inclusion criteria specified that all the participants were adults, most studies reported recruiting from a broad age range with the smallest age range within a study being 26 years [31]. Two studies [19,22] specified only that the participants were >18 years, and one [30] did not specify age range. 

In terms of the stated study aims, five studies specified weight loss [19,21,22,28,30], one aimed to limit weight gain [29] and one aimed to limit gestational weight gain [31]. Three studies stated the assessment of weight [25] or BMI [23,24] as part of their aim, while the remaining four studies did not mention weight or BMI in their aim, but did measure one or both of these outcomes [20,26,27,32]. The participant groups varied in their baseline BMI distribution. As might be expected, of the five studies aimed at weight reduction, four deliberately set out to recruit overweight or obese individuals and the mean baseline BMI for the participants in those studies was in the obese range [19,21,22,28]. The remaining study with weight loss as an aim, did not report the weight status at study entry [30]. The study by Loprinzi and colleagues aimed at weight gain prevention did not report baseline BMI, but instead reported categories of percent ideal body weight (IBW), with 36% of their participants more than 20% above IBW at baseline [29]. The study by Wolff and colleagues [31], aimed at restricting gestational weight gain, deliberately recruited obese women. Of the three studies that stated assessing the effect on weight or BMI as part of their aim, one [23] had participants with a BMI in the overweight range and the other [24] had participants in the healthy weight range. The study by Liu and colleagues [25] separated participants into two BMI categories. Those with a baseline BMI of 24 kg/m^2^ or above received weight loss advice, while those with a BMI below 24 kg/m^2^ were advised not to lose weight. For the four studies without stated aims around weight, two [26,27] had participants with a mean baseline BMI in the healthy weight range, while participants in the studies by Delahanty and colleagues [20] and the study by Huang and colleagues [32] had a mean baseline BMI in the overweight range. 

Weight was the most common form of anthropometric variable measured, being included in all 14 studies. Six of the studies [22,25,28,29,30,31] listed an anthropometric measure as the primary outcome variable. One study [24] listed anthropometry as a secondary outcome variable, and all the other studies measured anthropometric variables without further specification. Only one study used self-reported weight. The study by Wolff and colleagues examining gestational weight gain asked participants to self-report their weight prior to conception (the study end-point was measured by the research team) [31]. For all other studies, the research team measured weight at baseline and at study end points. Eight of the studies [19,21,22,23,24,25,26,32] used data on weight and height to calculate BMI. Waist circumference was reported as an outcome variable in four studies [19,21,22,25]. Two studies also reported data on percentage of body fat [19,25]. In terms of metabolic measures, four studies [22,26,31,32] collected blood glucose data with participants fasting at the time of collection. The study by Imai and colleagues [26] and the study by Huang and colleagues [32] also collected HbA1c. The study by Wolff and colleagues [31] collected serum insulin levels and conducted an oral glucose tolerance test. Two studies measured blood pressure [24,30]. Only three papers [22,28,29] reported powering the study for the primary outcome of weight change. While Rhodes and colleagues, [23] and Naldi and colleagues [21], also reported a sample size calculation, they did not reference an evidence source for their figures nor state the outcome measure upon which the calculation was powered. 

Participants received dietetic intervention for a variety of medical reasons including T2DM [22,26,32], T2DM prevention [25], hyperlipidemia [20,23,27], hypertension [24,30], overweight and obesity [19,28], limiting weight gain during pregnancy [31], preventing weight gain during chemotherapy for breast cancer [29] and psoriasis [21]. The dietitian interventions varied in terms of the number of consultations and their length, and the intervention duration. All but the study by Ramsay and colleagues [30] specified the total number of consultations, which varied from a minimum of one [24] to a maximum of 12 [26]. The minimum consultation number was seen in the study by Wong and colleagues [24] with a single dietitian session conducted just after allocation, and the maximum number of sessions was in the study by Ash and colleagues who used 11 [19] visits over a six-month period. The group mean of the total time spent in dietetic consultation was reported or could be calculated for seven studies [20,21,23,24,26,28,31] and ranged from 25 minutes [24] to 10 hours [31]. The intervention length ranged from 2 months to 12 months, with a median of 6 months. While the end points were generally measured at the end of intervention delivery, or shortly thereafter, two studies examined more distant end points. The study by Wong and colleagues [24] analyzed end-points 6 and 12 months after their single intervention session, and the study by Liu and colleagues [25] examined study endpoints at 12 months, some eleven months after the intervention contact with the dietitian. Three studies assessed effect maintenance by again collecting data 3 [31] or 6 months after the study end point [19,20].

The type of dietary advice provided by the dietitian in the intervention varied (shown in Table 2). Of the five interventions aimed at weight loss, four gave advice based on restricting energy intake, by specifying a set intake of 3300KJ [30], calculating a 15% decrease in caloric intake [22], setting energy intake at 80% of estimated requirements [21], or making individual prescriptions for an energy deficit to achieve a loss of 0.5–1 kg per week [19]. The study by Kesman and colleagues prescribed food portions rather than specifying energy and macronutrient intake [28]. Studies aimed at preventing weight gain incorporated dietary advice to limit weight gain to within five pounds [29] while a calculated energy increase was recommended in the study by Wolff and colleagues [31] to allow for fetal growth. Of the studies assessing effects on weight or BMI, Rhodes and colleagues [23] limited the proportion of energy supplied by fat and saturated fat [23], Wong and colleagues used the Dietary Approaches to Stop Hypertension (DASH) diet goals [24] and Liu and colleagues specified 10% caloric reduction to achieve weight loss but only in those with a BMI of 24 or above [25]. All participants in the Liu study received the same advice on macronutrient distribution (fat <30% calories, carbohydrate 55–65% calories and 20–30g of fiber/day. For the studies aimed at reducing blood lipid levels, Delahanty [20] used national (USA) cholesterol lowering guidelines, while Johnston and colleagues [27] stated only that the dietitian counselled on food planning, cooking methods and recipe modification. For the studies aimed at T2DM management, the study by Imai and colleagues described the dietary advice as being aimed at achieving dietary behavior change [26], while the study by Huang and colleagues [32] aimed to avoid excessive energy intake while following set macronutrient profiles.

Comparator groups were characterized as usual care, minimal care or control. Studies used this terminology in various ways, with some studies describing the control condition as the absence of any information [31] while others provided written information [19] or a general information session [21,25] under the heading of a control condition. The type of information provided is described in Table 2. The most frequently cited comparator was nutrition advice delivered by another health professional such as a doctor or nurse which was listed as the comparator in seven studies [20,21,23,24,26,29,30,32]. Brief oral information was provided by a study investigator in the case of the multinational study by Niswender and colleagues [22]. Other studies provided only written information to the control group [19,27,28]. One study used a control group that received no intervention [31].

### 3.2. Results of Included Studies

Six studies listed a positive effect for the intervention on weight and/or BMI [19,20,21,23,25,30,31]. For four of the other eight studies [19,22,24,27], while there was no between group difference, the dietitian intervention was as effective in achieving significant weight or BMI loss as usual care or brief advice [22,24] or written information [19,27]. The four remaining studies [26,28,29,32] found no significant changes in weight or BMI as a result of the dietitian interventions. 

It is important to consider these results in the context of the stated study aims of the individual studies. Of the five studies with weight loss as a stated study aim, there was an intervention effect for weight in the studies by Naldi and colleagues [21] (N = 303), and Ramsay and colleagues [30] (N = 49), but not in the studies by Kesman and colleagues [28] (N= 65), or Niswender and colleagues [22] (N = 611). The study by Ash and colleagues [19] (N = 119) showed a difference in weight change of 1.6 kg in favor of the individualized dietitian intervention at the six-month study end point, but did not report the statistical significance of this result. For the study by Liu et al. [25], there was an overall positive intervention effect, with the intervention group losing 0.57 kg more weight than the control group. However, the result was more pronounced for those who were overweight at baseline, where the intervention group lost 1.29 kg more weight than the control. Of the two studies that aimed to assess the effect of the nutrition care provided by a dietitian on BMI, Rhodes and colleagues [23] (N = 93) found an intervention effect for BMI, while Wong and colleagues [24] (N = 556) did not. For the four studies that reported on weight or BMI change but did not have this as a stated aim, the study by Delahanty and colleagues [20] (N = 90) found that the group receiving dietitian intervention lost weight whereas the control group did not change, but the studies by Johnston and colleagues [27] (N = 131), Huang and colleagues [32] (N = 154), and by Imai and colleagues [26] (N = 87) found no effect of the dietitian intervention. Of the two studies that aimed to prevent weight gain, the study by Loprinzi and colleagues (N = 109) [29] found no effect of the intervention above the control, while the study by Wolff and colleagues (N = 66) [31] found that the intervention was successful in achieving significantly less gestational weight gain than the control group. Of the four studies that included measures of glucose metabolism, three reported a positive effect of the intervention: the study by Huang and colleagues [32] for fasting plasma glucose; the study by Imai and colleagues [26] for HbA1c with T2DM patients; and that by Wolff and colleagues [31] for fasting serum insulin and glucose levels in obese pregnant women at 36 weeks gestation. The two studies measuring blood pressure found no effect of the dietitian intervention above usual care by a physician [24,30].

Three studies [19,20,31] included follow-up data months beyond the intervention end point (data not shown in table). Delahanty and colleagues [20] found that the intervention effect observed at six months was not maintained to 12 months, and the 2.6 kg weight loss observed at six months in the study by Ash and colleagues was reduced to a loss of 1.8 kg at 12 months compared to a weight gain of 0.5 kg by the control group [19]. In contrast, Wolff and colleagues found that the intervention effect observed at 36 weeks gestation was maintained to 4 weeks post-partum, with a difference of 6.9 kg (the intervention group weighed 4.5 kg less than they had at conception, while the control group weighed 2.4 kg more than they had at conception) [31]. 

### 3.3. Meta-Analysis

**Figure 2:**
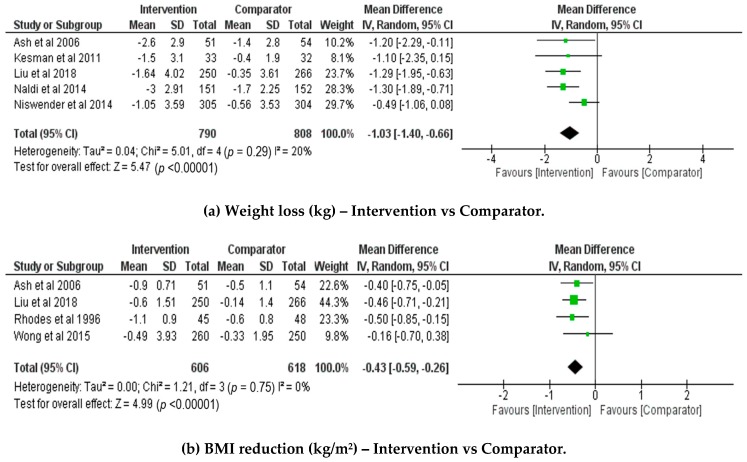
Forest plots showing the comparisons for individual dietetics counselling versus minimal or usual care from baseline to intervention end for (**a**) weight loss (kg) and (**b**) BMI (kg/m^2^).

Figure 2a illustrates the meta-analyses performed for the five studies with weight loss aims for which data could be extracted [19,21,22,25,28]. Figure 2b shows the meta-analysis for four studies of BMI change. As only two studies were aimed at weight gain prevention, no meta-analysis was performed on this outcome. Between-study heterogeneity was low, and not statistically significant for weight loss (I^2^ = 20%, *p* = 0.29) or BMI reduction (I^2^ = 0%, *p* = 0.75). The pooled mean difference, showing absolute change in values following intervention, favored individual dietetic consultations to induce both weight loss (pooled mean difference −1.03kg with 95% CI [−1.40, −0.66], *p* < 0.0001), and BMI reduction (pooled mean difference −0.43 kg/m^2^ with 95% CI [−0.59, −0.26], *p* < 0.00001). The squares in Figure 2 represent the weighted mean difference for each study. The size of the square represents the study weighting, with the larger squares corresponding to studies with a larger sample size. The diamond represents the pooled mean and 95% CI for all studies.

The sensitivity analysis for weight loss showed greater influence of the final two studies on the overall results. Removal of the study by Niswender and colleagues [22] from the analysis increased the effect size, with the results remaining statistically significant (pooled mean difference: −1.26kg; 95%CI [−1.65, −0.88], *p* < 0.00001; I^2^ = 0%, *p* = 0.99). Removal of the study by Liu and colleagues [25] decreased the effect size, with the results remaining statistically significant (pooled mean difference: −0.96kg with 95% CI [−1.41, −0.50], *p* < 0.0001; I^2^ = 28%, *p* = 0.24). Removal of the study by Naldi and colleagues [21] decreased the effect size, though the results remained significant (pooled mean difference: −0.93kg; 95%CI [−1.37 −0.48], *p* < 0.0001). Removal of either of these two studies, [22] or [25], individually, resulted in a reduction of the between-study heterogeneity (I^2^ = 0%, *p* = 0.99; or I^2^ = 28%, *p* = 0.24, respectively). However, removal of the study by Naldi et al. [21] resulted in no change to the between-study heterogeneity. The influence of these three studies on the pooled results is likely due to the large sample sizes and small measured error. Removal of any other study, individually, did not significantly influence the overall effect. The stability of the significant meta-analysis result despite the removal of studies with high weighting supports the effectiveness of dietetic consultations for weight loss.

The sensitivity analysis for BMI reduction showed greater influence of the third study on the overall results. Removal of the study by Liu and colleagues [25] decreased the overall effect size, while remaining statistically significant (pooled mean difference: −0.40 kg/m^2^ with 95% CI [−0.62, −0.18], *p* = 0.0005; I2 = 0%, *p* = 0.58). Removal of the study by Rhodes et al. [23] from the analysis decreased the overall effect size while also remaining statistically significant (pooled mean difference: −0.40 kg/m^2^; 95%CI [−0.60, −0.21], *p* < 0.0001; I2 = 0%, *p* = 0.61). Removal of any other study, individually, did not significantly influence the overall effect. The stability of the significant meta-analysis result, despite the removal of individual and highly weighted studies, supports the effectiveness of dietetic consultations in decreasing BMI.

## 4. Discussion

The current paper adds to the emerging literature on dietitian effectiveness and broadens the evidence base by focusing on weight management interventions. Despite the interest in evidence-based weight management, this is the first study to synthesize and meta-analyze data on the effectiveness of dietitians providing nutrition care for weight management in individualized consultations. While only six of the fourteen studies showed statistically significant intervention effects, a further four studies had interventions that were as favorable as the usual care control condition. Pooling of the data in meta-analyses showed that the dietitian intervention achieved an additional loss of weight and BMI when compared to usual care or written information. Given this is the first review to focus on weight and individualized dietitian consultations, our findings will be compared with systematic literature reviews of RCT interventions with a dietary change component aimed at weight control but not exclusively delivered by dietitians in individualized, face-to-face care.

While statistically significant, the mean change in weight and BMI due to the dietitian intervention found in the meta-analysis was relatively small and of limited clinical significance at just over one kilogram and 0.43 kg/m^2^, respectively. However, it is important to note that this was in addition to the weight and BMI lost through usual care, with the dietitian interventions achieving a total mean of 2 kg of weight loss. A systematic review by Moller and colleagues found that individualized nutrition therapy provided by a dietitian resulted in 2.1 kg more weight loss and 0.55 kg/m^2^ BMI loss than advice provided by other health professionals in patients with T2DM [11]. The same study also found that individualized nutrition therapy achieved superior results for HbA1c and LDL cholesterol levels. That review had similar advantages and limitations to the current review in terms of focusing on data from RCTs, but with relatively few (five) studies meeting the criteria for meta-analysis (one of which was the study by Huang and colleagues [32] in this review). Sun and colleagues [12] recently published their systematic review and meta-analysis of dietitian and non-dietitian delivered interventions aimed at preventing T2DM through weight loss. In amounts remarkably consistent with our study, they found that participants in the dietitian-delivered interventions lost a mean of 2.1 kg, which was 1 kg more than those in non-dietitian delivered interventions. 

It was unfortunately not possible to conduct a cost-effectiveness analysis for our review given the lack of cost data and the inability to calculate total time spent on each intervention. The review by Sun and colleagues also noted this limitation in the 69 studies included in their systematic review and meta-analysis, although they were able to calculate cost effectiveness for two of the dietitian intervention studies, with one study reported as $86.18/kg and the other at $325.10/kg [12]. While the ‘dose’ of the dietitian intervention could not be determined for all studies, it is important to note that none of the studies included in our review were of high intensity. As a comparison, a weight control study of five hours of dietitian time over the course of 12 months has been termed ‘relatively low-intensity’ [33], which would mean the studies included in this review could be categorized as ranging from low to moderate intensity. The lowest intensity study with a positive effect in our review [21] used an intervention that consisted of just 100 minutes delivered in five twenty-minute intervals resulting in a mean weight loss of 3 kg over 20 weeks compared with a mean weight loss of 1.7 kg by the control group. The highest stated intensity intervention that occurred in a study with a significant result was 10 hours of dietitian counselling delivered over six months for a 6.9 kg benefit over the control group [31]. Given this study aimed to limit gestational weight gain, the potential maternal and fetal health benefits of this moderate intensity intervention are significant. A systematic review, published in 2018 by Lamminpaa and colleagues, examined dietary interventions designed to limit or prevent gestational weight gain [34]. They found positive intervention effects for 10 of the 15 included studies, which included the study by Wolff and colleagues from our review [31]. The 15 studies varied in intervention intensity, and eight used dietary interventions that specified amounts of nutrients and/or energy, while the other seven studies reported using general dietary advice or national nutrition recommendations. The authors did not conduct a meta-analysis.

Despite our review having no date restrictions and comprised a synthesis of results from over four decades of RCT evidence, there were only fourteen studies reporting on anthropometric outcomes for individualized dietitian interventions, and only ten of those that had weight or BMI change as a stated aim of the study or primary outcome measure. This is perhaps surprising given that weight management is seen as one of the key roles of a dietitian and may reflect a lack of RCT research being conducted in this setting. Similarly, the review by Moller and colleagues included only five studies that measured the outcomes of BMI and HbA1c relevant to their population of people with T2DM [11]. It should be noted, however, that we deliberately excluded interventions that included dietitians as part of a multidisciplinary team, and those delivered by dietitians in an online format, which is likely to have limited the number of results, particularly in the most recent literature.

These types of studies were deliberately excluded for several reasons. While multidisciplinary teamwork is important, and frequently recommended as best practice, it is not always the method used in the primary health care setting. We also felt it was important to be able to elucidate the specific contribution made by dietitians to assist in workforce and resource allocation. Further, the evidence base on the role of dietitians as part of a multidisciplinary team has previously been synthesized in a systematic review by Tapsell and Neale [35]. They found that for the 16 RCT, pseudo-RCT and pre-post studies included in their review, interdisciplinary interventions were consistently successful in achieving change in weight, but not in other metabolic measures. The authors noted that many of the interventions were delivered by dietitians in combination with exercise physiologists and psychologists, rather than the professions that comprise the majority of the primary care workforce, doctors and nurses [35]. Similarly, the literature on weight control achieved by interventions delivered online has been previously synthesized [36,37]. Results of the most recent systematic review and meta-analysis conducted by Hutchesson and colleagues [37] found that eHealth interventions achieved weight loss of 2.7 kg more than the control group, while minimal interventions achieved a loss of 1.4 kg. 

The range in observed weight change results for the included studies could be explained by real differences or by study artefacts in this evidence base. Potential methodological design factors include: the majority of studies being of less than 12 months duration, lack of attention given to specifying a primary outcome or powering the study sufficiently for that outcome variable, lack of standardized endpoints and their measurement and/or reporting, variation in the control conditions used (from other health professionals delivering dietary advice, to written information to no intervention) and an overall lack of clear description of methods and results which we have reported previously [38]. Most studies did not separate the results by gender, and it is possible that one gender may respond better to individualized dietetic interventions that are traditionally delivered by women in this female-dominated profession. While the effects of participant gender have been examined in a systematic review of weight loss intervention studies [39], the impact of gender on outcomes from individualized dietetic consultations has not been studied.

Heterogeneity in the intervention design and in the dietary goals of the intervention also makes evidence base synthesis difficult. We could reasonably expect the observed result of an intervention of 10 hours duration delivered over the course of a pregnancy [31] having a superior effect than a one-off dietetic consultation of 20 minutes duration [24]. However, other results made less sense, such as the intervention totaling six hours delivered over one year having no effect [26], while a single consultation achieved one of the highest amounts of weight change over the same period [30]. The inability to calculate a total ‘dose’ of dietitian time for each of the studies limits the comparability, and future studies need to clearly report total time spent to allow cost-effectiveness calculations. Studies also need to detail the dietary goals and advice used in the interventions. Even when the dietary goal was weight loss, as in five of the studies, five different dietary modification methods were applied in pursuit of that goal.

ITT is the use of data for all randomized participants in the analysis for the primary outcome variable [40]. Despite the fact that it is a CONSORT standard for RCTs [41,42], not all studies reported intention-to-treat analyses. The limitation of relying on per-protocol analysis can be seen in the results of the study by Kesman and colleagues [28] who found a significant result for the participants completing the trial, which was no longer significant when analyzed using ITT principles. In contrast, the study by Naldi and colleagues had a higher number of participants and reported that they achieved similar results for the ITT and per protocol analyses [21]. It was more common for papers published after the year 2000 to use and specifically refer to ITT, with seven of the ten papers doing so. No papers published before 2000 reported ITT. A review of the use of ITT in reports in the top medical journals conducted by Bell and colleagues [43] found that use of the term ITT had increased since 2001, and lack of ITT reporting will hopefully be less of a limitation in future intervention studies. It should be noted that some studies that conducted per-protocol analyses had little missing data (for example, the study by Loprinzi and colleagues lost only 2 of 109 participants [29] and the study by Rhodes and colleagues lost 7 of 100 participants [23]).

## 5. Limitations

There were several limitations to the systematic review and meta-analyses. It is possible that the studies that involved individualized dietetic interventions were missed in the search if the term ‘dietitian’ was not used in describing the intervention. This highlights the importance of researchers providing a detailed intervention description and careful use of key words. By not having a date limit, studies that spanned four decades were compared which makes comparability difficult. Weight was not the primary study aim or outcome measure for all the included studies, but at least it was measured rather than self-reported (with the exception of the weight at conception in the gestational weight gain prevention RCT) [31]. A further limitation was that not all studies included similar populations, and they varied in baseline BMI. Eight of the studies [20,22,23,24,25,29,31,32] reported measuring some aspect of dietary intake which limits the assessment of intervention fidelity for the body of evidence. 

The meta-analysis is limited by the inclusion of studies with different comparator groups and the results should be interpreted with caution. These groups were not equal; the advice of an obesity researcher could reasonably be expected to have a greater effect than a ‘no information’ control. However, excluding these would have meant there were too few studies to conduct the analysis. As it is, there were only five studies eligible for the weight and BMI analyses respectively, which increases the risk of error and bias in the results. A further limitation is that not all studies included in the BMI meta-analysis conducted ITT analyses (although all studies included in the weight meta-analysis did). The study by Rhodes and colleagues [23] did not report using ITT, although the risk of bias from their inclusion in the meta-analysis is decreased by the fact that they reported only 7 dropouts out of 100. These factors largely reflect the usefulness of the current evidence and methodological improvements need to be applied to studies to improve the evidence base and practice guidelines for the effectiveness of dietitian consultations at achieving weight management.

## 6. Conclusions

The most common nutrition-related problem in the population is overweight and obesity, with dietitians being the main group responsible for dietary interventions to prevent and treat the problem. The main delivery vehicle for the management of overweight and obesity is the individualized consultation. Despite this, this is the first synthesis of the evidence that individualized consultations with a dietitian make a small but significant difference in weight control. Future RCTs to explore the influence of dietitian consultations on weight management are warranted. In particular, cost-effectiveness analyses will be important in determining the economic benefits of this modest weight loss.

## Figures and Tables

**Table 1:** Study selection criteria for the systematic review of the effectiveness of dietitians in weight control.

**Population:** Adult patients who have received an individual dietetic consultation within a primary health care setting
**Intervention:** Individualized nutrition care for weight management provided by a dietitian in primary health care
**Comparator:** No intervention (including pre-intervention); usual care (where patients received usual care from another health professional or health program); and minimal care (nutrition-related print material, or a once-off general nutrition seminar)
**Outcome:** Change in any anthropometric measure: weight (including gestational weight gain), Body Mass Index, skinfolds, waist circumference, waist-to-hip ratio
**Study design:** Systematic reviews of Randomized Controlled Trials (RCTs) and RCTs

**Table 2:** Details of the included randomized controlled trials (RCTs) assessing anthropometry: the measures and outcomes.

1st Author Year, Country	Study Aim	Participants		Anthropometry Measures Other Metabolic Measures ITT or Per-Protocol Analysis # and Study End Point	INT ERVENTION: Dietary Advice, Consultation Number and Period; Total Time N Completed	Comparator N Completed	Risk of Bias	Mean (SD) Change in wt (kg), BMI (kg/m^2^), WC (cm)	
		Recruitment Criteria	Baseline Characteristics *					INT	CON
**Studies citing decrease in weight or BMI as study aim or primary outcome variable**									
Ash 2006 Australia [19]	Compare group wt reduction intervention to individual dietetic care and written information.	>18 y; BMI ≥ 27 kg/m^2^; OPC patients without cognitive impairment.	INT N = 65; 16M; 49F Age = 48 (13) y BMI = 34.2 (5.9) kg/m^2^ CON N = 54; 12M; 42F Age = 47 (14) y BMI = 35.8 (6.2) kg/m^2^	Anthropometry: Wt, % body fat measured by trained researchers on bioelectrical impedance scales. BMI; WC (at umbilicus) Other metabolic: N/M # ITT (method unclear) used to analyze data at 6 months (N = 119)	Diet prescription aimed at wt loss of 0.5–1 kg/wk. 11 over 6 months; N/S. N = 49	Written information N = 24 (Group education INT (N = 29) N/A)	Unclear	Wt ^b^ −2.6 (4.0) BMI −0.9 (1.1) WC ^b^ −4.8 (7.5)	Wt ^b^ −1.0 (3.4) BMI −0.4 (1.4) WC ^b^ −4.6 (7.2)
Kesman 2011 USA [28]	Assess effectiveness of weight loss diet counselling in obese adults in medical primary care practice.	18-75y; Mayo OPC; BMI ≥30 to <40 kg/m^2^; without: cancer, pregnancy, AN, BN, psychiatric illness or surgery, gastric bypass, wt loss Tx.	INT N = 33; 13M; 20F Age = 55 (9.4) y Weight = 97.6 (12.8) kg CON N = 32; 12M; 20F Age = 56.3 (10.7) y Weight = 98.8 (12.5) kg	**Anthropometry**: % Wt change (primary) Wt collected on digital scales by researcher (shoes off) **Other metabolic**: N/M **ITT** (LOCF) used to analyze data at 6 months (N = 65) # **Per protocol analysis**: 65 at baseline; 42 at 6 months	Portion control plate: ¼ protein ½ vegetables; ¼ starch/grain 4 over 6 months (60 mins face to face + 3 phone); > 60 mins N = 19	Written information N = 23	High	Wt −1.0 (3.0)	Wt −0.5 (3.6)
Naldi 2014 Italy [21]	Assess dietary intervention plus physical exercise for weight loss on improving psoriasis in overweight or obese adults.	18–80 y; BMI ≥ 25 kg/m^2^; Hx chronic plaque psoriasis (PASI 10+); without: other psoriasis, weight loss Tx, pregnant/lactating, other chronic disease.	INT N = 151; 114M; 37F Age = 53 (16.7 IQR) y BMI = 30.8 (6.4IQR) kg/m^2^ CON N = 152; 101M, 51F Age = 53 (21 IQR) y BMI = 30.8 (6 IQR) kg/m^2^	**Anthropometry**: Wt and WC collected by trained researchers. BMI. **Other metabolic**: N/M **Primary**: PASI score change **ITT** (LOCF) used to analyze data at 20 weeks (N = 303) # **Per Protocol analysis**: 303 at baseline; 282 at 20 weeks	Week 1 to 12: EI: 0.8 x RMR; week 13–20: 1.0 x RMR. Fat = 30% EI, Carbohydrate = 55% EI, Protein = 15% EI 5 over 20 weeks (20 min each); 100 min N = 137	Control (15 min session advising wt reduction for psoriasis control) N = 145	Unclear	Wt ^a,b,c^ −3.0 (4.5) WC ^a,b,c^ −3.0 (5.0)	Wt ^b,c^ −1.7 (3.0) WC ^a,b,c^ −2.0 (3.5)
Niswender 2014 Multinational [22]	Determine impact of modest dietary intervention on weight change for overweight T2DM adults initiating insulin.	>18 y; BMI = 25–45 kg/m^2^; T2DM > 6m poorly controlled on metformin (HbA1c 7–9%); without: insulin, pregnancy, wt-affecting medications or conditions.	INT N = 306; 153M; 152F Age = 58.2 (9.7) y BMI = 34.4 (5.4) kg/m^2^ CON N = 305; 158M, 143F Age = 56.5 (10.0) y BMI = 34.3 (5.6) kg/m^2^	**Anthropometry**: Wt change (primary); BMI; WC. Measurements collected as part of study but details N/S **Other metabolic**: FPG, PPG, HbA1c **ITT** (LOCF) used to analyze data at 26 weeks (N = 611) #	Decrease caloric intake by 15%. 6 (3 face-to-face 3 by phone) over 22 weeks; N/S N = 246	Minimal care (basic lifestyle advice from the local investigator) N = 242	Unclear	Wt ^b^ −1.05 (3.59) WC ^b^ −1.79 (4.54)	Wt ^b^ −0.56 (3.53) WC ^b^ −1.02 (5.03)
Ramsay 1978 Scotland [30]	Compare efficacy of advice by diet sheet, doctor and dietitian on weight loss to reduce BP in BP clinic adults.	Age range NS; overweight on clinical judgment; no dietitian visit in 6 months prior, no special diet for medical reasons.	INT N = 15; Age = N/S BMI = N/S CON N = 20; Age = N/S BMI = N/S	**Anthropometry**: Wt change (primary) Measured by clinicians **Other metabolic**: DBP; SBP **Per Protocol analysis**: 67 at baseline; 49 at 12 months	3.3 MJ diet prescribed by dietitian. At least one over 12 months N/S N = 15	Minimal care (doctor advice to lose weight) N = 20 (Diet sheet N/A N = 14)	High	Wt ^a,c^ −5.10 (−15 to 0)	Wt ^c^ −2.15 (−9 to +5)^c^
**Studies citing effect on weight or BMI as part of study aim**									
Liu 2018 China [25]	Overall study aim: to assess extent to which a dietitian intervention can prevent T2DM development in normal wt and overweight women with GDM over 5 years. Aim of this paper: to analyze weight change results after first year of the study.	24–49 y; GDM in preceding 4 years (diagnosed by OGTT using WHO criteria) without medications to influence BGLs; chronic disease; current or planned pregnancy.	INT N = 586 Age = 32.3 (3.4) y BMI < 24 kg/m^2)^ (NW) = 57.3% BMI ≥ 24 kg/m^2^ (OW) = 42.7% CON N = 594 Age = 32.4 (3.6) y BMI < 24 kg/m^2^ (NW) = 57.3% BMI ≥ 24 kg/m^2^ (OW) = 42.7%	**Anthropometry**: Wt change, BMI; % body fat, WC. **Other metabolic**: N/A **ITT** (missing values imputed) (**N = 1180**) # **Per Protocol analysis**: 1180 at baseline; 930 at 12 months.	**BMI < 24 kg/m^2^** Fat < 30% EI, Carbohydrate 55–65% EI, 20–30 g Fibre/day prescribed by dietitian + 5 day diet and written handbook. **BMI ≥ 24 kg/m^2^**: as above plus 10% calorie reduction to lost 5–10%wt. 6 over first 4 weeks N/S N = 460	Usual care: Two diet education sessions on T2DM prevention. N = 470		**All** Wt ^a^ −0.64 (3.29) BMI ^a^ −0.25 (1.22) **NW** Wt −0.10 (2.37) BMI −0.004 (0.88) **OW** Wt ^a^ −1.64 (4.02) BMI ^a^ −0.60 (1.51)	**All** Wt −0.07 (2.93) BMI −0.03 (1.13) **NW** Wt −0.15 (2.20) BMI −0.06 (0.85) **OW** Wt −0.35 (3.61) BMI −0.14 (1.40)
Rhodes 1996 USA [23]	Compare effect of OPC dietitian with usual care on nutrition, BMI, and lipids in initial hypercholesterolemia management.	30–65 y; LDL-C >4.14 mmol/L or >3.36 mmol/L + other risk factors; without: T2DM, pregnancy, liver conditions, triglycerides >2.82 mmol/L, lipid lowering meds in past 2/12.	INT N = 45; Age = 47.5 (9) y Weight = N/S BMI = 28.1 (4.2) kg/m^2^ CON N = 48 Age = 47.5 (9) y Weight = N/S BMI = 28.3 (4.3) kg/m^2^	**Anthropometry**: BMI Method of collecting weight and height N/S **Other metabolic**: N/M **Per Protocol analysis**: 100 at baseline; 93 at 3 months	F ≤ 30% EI; Saturated Fatty Acids ≤ 10% EI, <300mg cholesterol. 3 over 7 weeks (Initial 60 min, reviews 30 min); 2 hours. N = 45	Minimal care (10 minutes of advice from Physician/nurse) N = 48	Unclear	BMI ^a,b^ −1.1 (0.9)	BMI ^b^ −0.6 (0.8)
Wong 2015 China [24]	Compare DASH diet and dietitian counselling with usual care on BP, fasting lipid profile, and BMI.	40–70 y; newly diagnosed grade I hypertension; without: medical conditions requiring dietary control, antihypertensive medication;	INT N = 281; 131M, 150F Age = 55.4 (5.6) y BMI = 24.17 (2.83) kg/m^2^ CON N = 275; 142M, 133F Age = 54.9 (5.2) y BMI = 24.23 (3.06) kg/m^2^	**Anthropometry**: BMI (secondary). Wt measured by clinicians in indoor clothing and height on a wall-mounted stadiometer. **Other metabolic**: SBP, DBP (primary) **ITT** (LOCF) used to analyze data at 12 months (N = 556) #	DASH diet goals for food groups. 1 x 25 min over 6 months 25 min N = 243	Usual care (physician) N = 242	Unclear	BMI ^b^ −0.49 (3.93)	BMI −0.33 (1.95)
**Studies citing weight gain prevention as study aim**									
Loprinzi 1996 USA [29]	Can dietitian counselling prevent wt gain in women receiving adjuvant systemic chemotherapy for resected breast cancer.	26–57 y; Women on chemotherapy post breast resection; without: special diet needs, wt >20% below IBW, conditions/medications causing wt gain or fluid retention.	INT N = 54; 54F Age = 43 y BMI = N/S CON N = 53; 53F Age = 43 (26–57) y BMI = N/S	**Anthropometry**: Wt change (primary). Measurements collected as part of study but details N/S **Other metabolic**: N/M **Per protocol analysis**: 109 at baseline; 107 at 6 months	Diet to limit wt gain to 5lb or less. 3 over 6 months; N/S N = 54	Usual care (physician/nurse advice to prevent weight gain) N = 53	High	Wt ^c,d^ +2.0	Wt ^c,d^ +3.5
Wolff 2008 Denmark [31]	Investigate if obese women can restrict GWG and pregnancy-induced increases in insulin, leptin, and glucose.	19–45 y; BMI ≥ 30 kg/m^2^; singleton pregnancy; non-smokers; without complications affecting foetal growth.	INT N = 23; 23F Age = 28.7 (4) y BMI = 34.9 (4) kg/m^2^ CON N = 27; 27F Age = 30.7 (5) y BMI = 34.6 (3) kg/m^2^	**Anthropometry**: GWG (primary): Wt measured by researchers at 36 weeks gestation— self-reported wt at conception **Other metabolic**: Fasting: Insulin; glucose, OGTT **Per Protocol analysis**: 66 at baseline; 50 at 36 weeks gestation	Total Energy Requirement = Basal Metabolic Rate X 1.4; Carbohydrate = 50–55% EI; Protein = 15–20% EI; Fat = 30% EI. 10 x 60 min over 24 weeks; 10 hours. N = 23	Control (no advice on diet) N = 27	High	Wt ^a^ +6.6(5.5)	Wt +13.3 (7.5)
**Studies that measured weight or BMI without stating them in study aim or as primary or secondary outcome variables**									
Delahanty 2001 USA [20]	To compare impact of cholesterol lowering protocol by dietitian with physician advice.	21–65 y; cholesterol 5.2–8.84mmol/L; without: dietitian contact in 12 months, medical conditions/meds influencing lipids.	INT N = 45; 30M, 15F Age = 49 (10) y Weight = 79.6 (15.4) kg CON N = 45; 30M, 15F Age = 49 (9) y Weight = 83.2 (15.0) kg	**Anthropometry**: Wt change measured to nearest 0.1 kg by trained researchers **Other metabolic**: N/M **ITT** (method unclear) used to analyze data at 6 months (N = 90) #	NCEP cholesterol lowering protocol. 4 over 6 months (90 min in first 3 months and 30 min in months 4 to 6. 2 hours. N = 44	Usual care (physician advice) N = 44	Unclear	Wt ^a^ −1.9 (21.2)	Wt 0 (8.08)
Huang 2010 Taiwan [32]	Are T2DM patients who receive dietitian consultations more likely to follow glycaemic control diet.	30–70 y; diagnosed T2DM; without pregnancy, dialysis, amputation, blindness, cancer or cardiovascular disease.	INT N = 75; 29M, 46F Age = 56.6 (8.0) y BMI = 25.7 (3.2) kg/m^2^ CON N = 79; 38M, 41F Age = 56.9 (7.5) y BMI = 27.0 (4.7) kg/m^2^	**Anthropometry**: BMI changeWeight and height measurement technique N/S **Other Metabolic**: FPG; HbA1c (primary) **Per protocol analysis**: 39 dropouts and only analyzed the 154 analyzed at 12 months	Avoid excessive EI. Carbohydrate = 50–60% EI; Protein = 15–20% EI; Fat = 25–30% EI. 4 (30–60 mins each) over 12 months. 2–4 hours N = 57	Usual care (summary of basic dietary principles by nurses) N = 58	High	BMI 3.3 (1.2)	BMI 0.2 (1.5)
Imai 2008 Japan [26]	Investigate effect of individual dietetic counselling on glycaemic control in T2DM patients	36–80 y; diagnosed T2DM; without: significant comorbidity.	INT N = 29; 13M, 16F Age = 62.0 (10.9) y BMI = 23.8 (4.1) kg/m^2^ CON N = 30; 14M, 16F Age = 64.3 (10.7) y BMI = 23.6 (2.9) kg/m^2^	**Anthropometry**: BMI change Weight and height measurement technique N/S **Other Metabolic**: FPG; HbA1c (primary) **ITT not stated** but no dropouts reported: 77 at baseline and 12 months	General diet advice. 12 (20–30 mins each) over 12 months; 4–6 hours. N = 29	Usual care (brief advice by Dr/Nurse) N = 30 (Food provision INT N/A N = 18)	High	BMI −0.3 (1.98)	BMI 0.3 (1.51)
Johnston 1995 Australia [27]	Compare efficacy of three diet and lifestyle interventions in lowering plasma lipids.	24–81 y; BMI > 20 kg/m^2^; TC 5.5–8.0 mmol/L; without: T2DM, Coronary Artery Disease, uncontrolled hypertension, pregnancy, appetite suppressants, lipid lowering meds.	INT N = 44; 13M, 16F Age = 56 (N/S) y BMI = 24.2 (22.7–26.4 IQR) kg/m^2^ CON N = 47; 14M, 16F Age = 56 (N/S) y BMI = 25.1 (22.3–26.3IQR) kg/m^2^	**Anthropometry**: Wt change Measurements collected as part of study but details N/S **Other metabolic**: N/M **Per protocol analysis**: 179 at baseline; 131 at 6 months	Diet change strategies: food planning, cooking methods, recipe modification. 3 over unstated period; N/S N = 44	Minimal care (written information) N = 47 (Group counselling INT N/A N = 40)	High	Wt ^b,c^ −1.0 (−3.0 to 0.0)	Wt ^c^ −1.0 (−2.0 to +1.0)

* BMI is reported where stated; in the absence of baseline BMI report, weight is included instead. # indicates ITT values reported in the results column. ^a^ Presence of statistically significant difference in group mean outcome for intervention v control. ^b^ presence of statistically significant difference from baseline for both groups, but no significant difference between the two conditions. ^c^ Data reported as median and IQR or range. ^d^ Error not reported by authors. **Abbreviations used in table:** BMI = Body Mass Index; BP = Blood Pressure; CON = control; DASH = Dietary Approaches to Stop Hypertension; DBP= Diastolic Blood Pressure; FPG = fasting plasma glucose; F = female; GWG= Gestational Weight Gain; HbA1c = Glycolated Haemoglobin; Hx = History; INT = Intervention; ITT = Intention-to-treat; LDL-C = Low density Lipoprotein Cholesterol; LOCF = Last Observation Carried Forward; mins = minutes; N/A = not analyzed; N/M = Not measured; N/S = not stated; NCEP = National Cholesterol Education Program; NW = Normal weight; OGTT = Oral Glucose Tolerance Test; OW = overweight; PASI= Psoriasis Area Severity Index; RMR = Resting Metabolic Rate; SBP = systolic blood pressure; T2DM= Type 2 Diabetes Mellitus; w = weeks; WC= waist circumference; Wt = Weight; y = years.

## References

[1] Roberto C.A., Swinburn B., Hawkes C.P., Huang T.T.K., Costa S.A., Ashe M., Zwicker L., Cawley J.H., Brownell K.D. (2015). Patchy progress on obesity prevention: Emerging examples, entrenched barriers, and new thinking. Lancet.

[2] World Health Organization (2016). Global Report on Diabetes.

[3] Dietz W.H., Baur L.A., Hall K., Puhl R.M., Taveras E.M., Uauy R., Kopelman P. (2015). Management of obesity: Improvement of health-care training and systems for prevention and care. Lancet.

[4] Bleich S.N., Bandara S., Bennett W., Cooper L.A., Gudzune K.A. (2015). Enhancing the role of nutrition professionals in weight management: A cross-sectional survey. Obesity.

[5] Lacey K., Pritchett E. (2003). Nutrition care process and model: Ada adopts road map to quality care and outcomes management. J. Am. Diet. Assoc..

[6] Raynor H.A., Champagne C.M. (2016). Position of the academy of nutrition and dietetics: Interventions for the treatment of overweight and obesity in adults. J. Acad. Nutr. Diet..

[7] BDA Obesity Specialist Group (2018). Dietetic Obesity Management Interventions in Adults: Evidence Review & Clinical Application.

[8] National Institute for Clinical Excellence (NICE) (2006). Obesity: Guidance on the Prevention, Identification, Assessment and Management of Overweight and Obesity in Adults and Children.

[9] Scottish Intercollegiate Guidelines Network (SIGN) (2010). Management of Obesity: A National Clinical Guideline.

[10] Academy of Nutrition & Dietetics (2012). Evidence Analysis Manual: Steps in the Academy Evidence Analysis Process.

[11] Moller G., Andersen H., Snorgaard O. (2017). A systematic review and meta-analysis of nutrition therapy compared with dietary advice in patients with type 2 diabetes. Am. J. Clin. Nutr..

[12] Sun Y., You W., Almeida F., Estabrooks P., Davy B. (2017). The effectiveness and cost of lifestyle interventions including nutrition education for diabetes prevention: A systematic review and meta-analysis. J. Acad. Nutr. Diet..

[13] Mitchell L., Ball L., Ross L., Barnes K., Williams L. (2017). Effectiveness of dietetic consultations in primary health care: A systematic review of randomized controlled trials. J. Acad. Nutr. Diet..

[14] Higgins J.P.T., Green S. (2011). Cochrane Handbook for Systematic Reviews of Interventions, Version 5.1.0. http://handbook.cochrane.org.

[15] (2014). Review Manager 5. RevMan.

[16] Sulivan L., LaMorte W. Interquartile Range (iqr). http://sphweb.bumc.bu.edu/otlt/mph-modules/bs/bs704_summarizingdata/bs704_summarizingdata7.html.

[17] Hozo S., Djulbegovic B., Hozo I. (2005). Estimating the mean and variance from the median, range, and the size of a sample. BMC Med. Res. Methodol..

[18] DerSimonian R., Kacker R. (2007). Random-effects model for meta-analysis of clinical trials: An update. Contemp. Clin. Trials.

[19] Ash S., Reeves M., Bauer J., Dover T., Vivanti A., Leong C., O’Moore Sullivan T., Capra S. (2006). A randomised control trial comparing lifestyle groups, individual counselling and written information in the management of weight and health outcomes over 12 months. Int. J. Obes..

[20] Delahanty L.M., Sonnenberg L.M., Hayden D., Nathan D.M. (2001). Clinical and cost outcomes of medical nutrition therapy for hypercholesterolemia: A controlled trial. J. Am. Diet. Assoc..

[21] Naldi L., Conti A., Cazzaniga S., Patrizi A., Pazzaglia M., Lanzoni A., Veneziano L., Pellacani G. (2014). Diet and physical exercise in psoriasis: A randomized controlled trial. Br. J. Dermatol..

[22] Niswender K., Piletic M., Andersen H., Conradsen Hiort L., Hollander P. (2014). Weight change upon once-daily initiation of insulin detemir with or without dietary intervention in overweight or obese insulin-naive individuals with type 2 diabetes: Results from the diet trial. Diabetes Obes. Metab..

[23] Rhodes K.S., Bookstein L.C., Aaronson L.S., Mercer N.M., Orringer C.E. (1996). Intensive nutrition counseling enhances outcomes of national cholesterol education program dietary therapy. J. Am. Diet. Assoc..

[24] Wong M.C., Wang H.H., Kwan M.W., Fong B.C., Chan W.M., de Zhang X., Li S.T., Yan B.P., Coats A.J., Griffiths S.M. (2015). Dietary counselling has no effect on cardiovascular risk factors among chinese grade 1 hypertensive patients: A randomized controlled trial. Eur. Heart J..

[25] Liu H., Wang L., Zhang S., Leng J., Li N., Li W., Wang J., Tian H., Qi L., Yang X. (2018). One-year weight losses in the tianjin gestational diabetes mellitus prevention programme: A randomized clinical trial. Diabetes Obes. Metab..

[26] Imai S., Kozai H., Matsuda M., Hasegawa G., Obayashi H., Togawa C., Yamamura T., Watanabe K., Miyatani S., Yoshikawa T. (2008). Intervention with delivery of diabetic meals improves glycemic control in patients with type 2 diabetes mellitus. J. Clin. Biochem. Nutr..

[27] Johnston H.J., Jones M., Ridler-Dutton G., Spechler F., Stokes G.S., Wyndham L.E. (1995). Diet modification in lowering plasma cholesterol levels. A randomised trial of three types of intervention. Med. J. Aust..

[28] Kesman R.L., Ebbert J.O., Harris K.I., Schroeder D.R. (2011). Portion control for the treatment of obesity in the primary care setting. BMC Res. Notes.

[29] Loprinzi C.L., Athmann L.M., Kardinal C.G., O’Fallon J.R., See J.A., Bruce B.K., Dose A.M., Miser A.W., Kern P.S., Tschetter L.K. (1996). Randomized trial of dietician counseling to try to prevent weight gain associated with breast cancer adjuvant chemotherapy. Oncology.

[30] Ramsay L.E., Ramsay M.H., Hettiarachchi J., Davies D.L., Winchester J. (1978). Weight reduction in a blood pressure clinic. Br. Med. J..

[31] Wolff S., Legarth J., Vangsgaard K., Toubro S., Astrup A. (2008). A randomized trial of the effects of dietary counseling on gestational weight gain and glucose metabolism in obese pregnant women. Int. J. Obes..

[32] Huang M.C., Hsu C.C., Wang H.S., Shin S.J. (2010). Prospective randomized controlled trial to evaluate effectiveness of registered dietitian-led diabetes management on glycemic and diet control in a primary care setting in taiwan. Diabetes Care.

[33] Williams L., Hollis J., Collins C., Morgan P. (2014). Can a relatively low-intensity intervention by health professionals prevent weight gain in mid-age women? 12-month outcomes of the 40-something randomised controlled trial. Nutr. Diabetes.

[34] Lamminpää R., Vehviläinen-Julkunen K., Schwab U. (2018). A systematic review of dietary interventions for gestational weight gain and gestational diabetes in overweight and obese pregnant women. Eur. J. Nutr..

[35] Tapsell L., Neale E. (2016). The effect of interdisciplinary interventions on risk factors for lifestyle disease: A literature review. Health Educ. Behav..

[36] Neve M., Morgan P., Collins C.E. (2010). Effectiveness of web-based interventions in achieving weight loss and weight loss maintenance in overweight and obese adults: A systematic review with meta-analysis. Obesity Rev..

[37] Hutchesson M.J., Rollo M.E., Krukowski R., Ells L., Harvey J., Morgan P.J., Callister R., Plotnikoff R., Collins C.E. (2015). Ehealth interventions for the prevention and treatment of overweight and obesity in adults: A systematic review with meta-analysis. Obes. Rev..

[38] Ball L., Sladdin I., Mitchell L., Barnes K., Ross L., Williams L. (2018). Quality of development and reporting of dietetic intervention studies in primary care: A systematic review of randomised controlled trials. J. Human Nutr. Diet..

[39] Williams R.L. (2015). Effectiveness of weight loss interventions—Is there a difference between men and women: A systematic review sex differences in men and women. Obes. Rev..

[40] Joshi M., Royuela A., Zamora J. (2013). Proper analysis in clinical trials: How to report and adjust for missing outcome data. BJOG Int. J. Obstet. Gynaecol..

[41] Begg C., Cho M., Eastwood S., Horton R., Moher D., Olkin I., Pitkin R., Rennie D., Schulz K.F., Simel D. (1996). Improving the quality of reporting of randomized controlled trials: The consort statement. JAMA.

[42] Moher D., Hopewell S., Schulz K.F., Montori V., Gotzche P., Devereaux P.J., Elbourne D., Egger M., Altman D. (2010). Consort 2010 explanation and elaboration: Updated guidelines for reporting parallel group randomised trials. Br. Med. J..

[43] Bell M.L., Fiero M., Horton N.J., Hsu C.-H. (2014). Handling missing data in rcts; a review of the top medical journals. BMC Med. Res. Methodol..

